# Possession in Football: More Than a Quantitative Aspect – A Mixed Method Study

**DOI:** 10.3389/fpsyg.2019.00501

**Published:** 2019-03-18

**Authors:** Claudio A. Casal, M. Teresa Anguera, Rubén Maneiro, José L. Losada

**Affiliations:** ^1^Department of Science of Physical Activity and Sport, Catholic University of Valencia San Vicente Mártir, Valencia, Spain; ^2^Department of Social Psychology and Quantitative Psychology, Institute of Neurosciences, University of Barcelona, Barcelona, Spain; ^3^Department of Science of Physical Activity and Sport, Pontifical University of Salamanca, Salamanca, Spain; ^4^Department of Social Psychology and Quantitative Psychology, University of Barcelona, Barcelona, Spain

**Keywords:** performance analysis, football, possession, UEFA Euro France, observational methodology, mixed methods

## Abstract

The aim of the present study was to identify and differentiate the factors that determine the possession times of successful and unsuccessful elite football teams, with the purpose of identifying a more effective possession model. For this, match corresponding to the round of eighth-finals, quarter-finals, semi-finals and final of the 2016 UEFA Euro France in which 2,636 offensive sequences occurred, were analyzed. Video recordings of matches were analyzed and coded post-event using systematic observation. The performance indicators recorded and analyzed were: phase; match period; type of start-up; interaction context; intention; field zone; possession time, passes, attack outcome; match status and final outcome. An ANOVA was performed to analyze data in order to study the influence of a set of variables. A Box–Cox transformation was applied on the variable explained to achieve normal conditions. A study of the main effects and significant interactions was also carried out, complemented with a set of predictions with the variables that were more significant. It is hypothesized that possession analysis from a *mixed methods* perspective will identify a more effective offensive playstyle. Results show how, in successful teams, possession time is influenced by: Type of start-up, intention and field zone. On the other hand, in unsuccessful teams, possession time is determined fundamentally by intention and match status. In terms of the results of the predictive models, in the case of successful teams, they will have longer possessions in the offensive zone with the score in favor and, in the defensive zone with a draw score, in both situations, initiated with the intention of progressing by means of a transition. For unsuccessful teams, possessions will be of longer duration in the defensive zone with a draw score, regardless of the type of start-up and, in the offensive zone, losing and initiating the play by means of a set ball action and winning by means of a transition. Results obtained in this work identify key factors that determine possession time in teams and allow to differentiate the possessions of successful and unsuccessful teams, identifying a more effective ball possession model. This information can be used to design a possession model with greater probabilities of success and increase the offensive performance of teams.

## Introduction

Ball possession, in recent years, has acquired transcendental importance in the offensive game model of many football teams. This circumstance was mainly caused by the success of teams such as the FC Barcelona, Manchester City, FC Bayer München or the Spanish and German national teams. All these teams are characterized by an offensive game model, based on the initiative of the game, through ball possession. Numerous previous works have confirmed that it is a performance indicator that makes it possible to differentiate high-level teams. Grant et al. (1999b) analyzed the 1998 World Cup, concluding that greater ball possession is linked to the team success. The work of Hook and Hughes (2001) showed how successful teams in the UEFA Champions League, World Champions and Europa Cup maintained longer possessions than unsuccessful teams. Bloomfield et al. (2005) reported that the three best teams of the English Premier League in the 2003–2004 season (Chelsea FC, Manchester United FC and Arsenal FC), maintained longer possession time than their opponents. Jones et al. (2004) found significant differences in possession time between successful and unsuccessful teams in the English Premier League. Carling et al. (2005) analyzed the same competition but in the 1996–1997 season, obtained the same results. Casal et al. (2015) analyzed Euro 2008, concluding that a longer duration of the offensive phase predicts a greater success of it. The analysis of possession in the 2016 UEFA Euro France made in the work of Casal et al. (2017), also corroborate the close relationship between longer possession time and team success.

In previous works quantitative analysis of possession are carried out, arriving in some cases to identify the zone in which it takes place (Casal et al., 2017), but in none of them the factors that modulate team possession time are identified, nor are they compared to acknowledge if these are the same in successful and unsuccessful teams. Therefore, a study that allows to create relationships between the quantitative and qualitative aspects of possession is justified, and that is not limited to describing and quantitatively comparing team possessions, but that tries to identify which are the performance indicators related to possession time and, describe an offensive playstyle that allows to guarantee greater longer possessions. Unlike most of the previously mentioned works, we intend to perform a novel analysis, from a *mixed methods* perspective. In this work, in addition to a quantitative analysis, we intend to carry out a qualitative analysis to study the quality of team possessions. The study of possession quality must be undertaken from a qualitative perspective, and the ideal option is systematic observation (Anguera et al., 2000, 2017), which guarantees a perfect balance between flexibility and rigor, and which must be integrated with conventional quantitative information in the study of possessions. Thus, this study is considered from the *mixed methods* perspective, which implies a novel treatment of possessions, which were usually studied only from a quantitative perspective, taking into account parameters obtained in many cases through computer programs oriented exclusively to a description of the competitions from element frequency (serves, shots on goal, penalties, etc.) of different nature along the game sets. We start from the *mixed methods* perspective because it represents a third emerging paradigm of research (Johnson et al., 2007) that offers an alternative to purely qualitative or quantitative studies which has expanded rapidly over the last two decades (Tashakkori and Teddie, 1998, 2003, 2010).

Through the application of this methodology we will try to achieve the following objectives: determining possession times of successful and unsuccessful teams, identifying performance indicators that influence possession times in both groups of teams, describing differences between possession patterns of successful and unsuccessful teams and, finally, find more effective possession models. Our hypothesis is that possession analysis from the *mixed methods* perspective will make possible to identify a playstyle that guarantees a more effective possession of the ball. If the hypothesis is confirmed, obtained results can be used by technicians and players to model training and competitions, allowing to increase the offensive performance of teams.

## Materials and Methods

### Observational Design

The specific design corresponding to this systematic observation, according to Anguera et al. (2011), is a combination of a nomothetic/puntual/multidimensional (N/P/M) and nomothetic/follow up/multidimensional (N/F/M) design. The reason is that some teams (nomothetic) are recorded in several matches with different opposite team (puntual), but also that these teams participate in the rounds until the final match (follow up); in all matches we have considered several performance indicators and time measures (multidimensional). Moreover, the recording used an intrasessional follow-up (frame-by-frame analysis of different matches), and was captured using the *ad hoc* observation instrument in different matches. Data analyzed is of type IV (Bakeman, 1978).

### Participants

To control some of the situational variables that can potentially affect tactical and strategic team behavior, such as quality or level of opposing teams and the match location (Kormelink and Seeverens, 1999; Carling et al., 2005), 14 teams and 12 matches corresponding to the round of eighth-finals, quarter-finals, semi-finals and final of the 2016 UEFA Euro France have been selected in which 2,636 offensive sequences occurred. Belgium, Croatia, Eire, England, France, Germany, Hungary, Iceland, Italy, Northern Ireland, Portugal, Slovakia, Spain, and Wales were the teams analyzed.

Three games Switzerland vs. Poland; Poland vs. Portugal and Germany vs. Italy have been excluded from the analysis since the match outcome was a draw having in account regular time and extensions, which makes impossible to label the teams as successful or unsuccessful.

This sample ensures that all matches are played on neutral ground, the teams have a similar level and, by eliminating the games of the group phase, we also make sure that the teams look for the victory in their matches, since defeat will mean elimination. In the group phase matches, it may happen that some team is more interested in drawing or losing any of their matches, to avoid a particular opponent in the following phases, this would lead to incorrect results in the study.

### Observational Instrument

Four national coaches and experts in football research designed an *ad hoc* observation instrument combining a field format and category systems (Anguera et al., 2018a,b) was created (Table 1). Indicators that represent the qualitative side of this study have been selected based on previous studies (Casal et al., 2015, 2016, 2017), which have demonstrated their relationship with team performance.

**Table 1:** Observational instrument (field format combined with category systems).

	Criterion	Definition	Categories
Identification	Team	Analyzed team	
	Phase	Match classification phase	o: round of 16
			c: quarterfinals
			smf: semifinal
			f: final
Classification	Final Outcome	Final match result regardless of attack sequence	w: win
			d: draw
			l: loss
Performance indicators	Match period	Part of the match in which the attack sequence was collected	ft: first time
			st: scond time
	Type of start-up	Way to start the attack sequence	sp: set pieces
			t: dynamic transition
	COI	Start interaction context	ar: advanced versus delayed
			am: advanced versus average
			aa: advanced versus advanced
			mm: average versus average
			mr: average versus delayed
			ma: average versus advanced
			ra: delayed versus advanced
			rm: delayed versus average
			pa: goalkeeper versus advanced
	Intention	Observed team intention when recovering the ball	p: progress
			k: keep
	Passes	Total observed passes that the team’s attack sequence had	Numeric
	Match Status	Team’s partial marker observed in the attack sequence	wn: winning
			dr: drawing
			ls: losing
Possession	MD	Time in SECONDS that the observed team keeps the ball in its DEFENSIVE zone	Seconds
	MO	Time in SECONDS that the observed team keeps the ball in its OFFENSIVE zone	Seconds
	ZC	Area in which the ball stayed longer in each offensive sequence	1: middle defensive zone
			2: middle offensive zone offensive
	Possession time	Total time the possession lasted (MD + MO)	Seconds
Outcome	Attack outcome	Final result of the team’s observed attack sequence	goal: goal
			sh: shot
			sta: sent to area
			ne: no success

Data were recorded and coded in the LINCE software program (Gabin et al., 2012), and the MASS packages have been used (Venables and Ripley, 2002), and CAR (Fox and Weisberg, 2011) from the R software (v. 3.4.1).

### Procedure

Matches were recorded from TV emitted images and were registered and analyzed post-event. Because the video recordings were public, confidentiality was not an issue and authorization was not required from the players observed or their representatives. Furthermore, the information cannot be considered either personal or intimate, as the research consisted solely of naturalistic observations in public places, and it was not anticipated that the recordings would be used in a manner that could cause personal harm (The American Psychological Association’s [APA’s], 2010). No experimental analysis involving human studies is performed in the study. Also, according to The Belmont Report (1978) the use of public images for research purpose does not required informed consent or the approval of an ethical committee. An ethics approval was therefore not required as per applicable institutional and national guidelines. Criteria used for the division of the teams into two groups, successful and unsuccessful, has been the outcome of the match (Lago-Peñas et al., 2010), excluding penalties. This way, all the teams that won their matches during reglementary time or extensions were classified as successful and teams who lost their matches as unsuccessful.

### Data Quality Control

To try to ensure data reliability, all matches were registered and analyzed by four observers, all of them national soccer coaches with more than 10 years of experience in the field of training, teaching and research in football through observational methodology. In addition, the following training process was carried out: First, eight observing sessions were conducted on teaching the observers following the Losada and Manolov (2015) criteria and applying the criterion of consensual agreement (Anguera, 1990) among observers, so that recording was only done when agreement was produced. To ensure inter-reliability consistency of the data (Berk, 1979; Mitchell, 1979) the Kappa coefficient was calculated for each criterion, it revealed a strong agreement between observers, which means high reliability (0.92), taking Fleiss (1981) as a reference, who establishes a classification for the Kappa values where it characterizes as regular values found between 0.40 and 0.60, good between 0.60 to 0.75 and excellent above 0.75. Moreover, the procedure was repeated after 2 weeks (to exclude any learning effects) to check intraobserver reliability (Mitchell, 1979).

### Statistical Analysis

A complete factorial design was applied to verify which were the factors that most influenced “Possession time” in successful and unsuccessful teams. The analysis of the variables and their interactions was carried out using the ANOVA technique. The residuals conditions were verified to check that normality conditions are met. In the case of non-compliance, a transformation of the response variable, “Total possession time” was performed, using a Box–Cox transformation where the parameter λ was estimated, by maximum likelihood.

After adapting the regression model and checking the adjustment to normality by calculating the Shapiro–Wilk statistic, the main effects and interaction relationships between the model’s significant variables were calculated. Finally, a set of predictions was calculated between the interactions that were significant, accompanied by their graphic representation, to compare successful and unsuccessful teams.

## Results

Analysis started with data selection and filtering, using the variables: *Type of start-up*, *Intention*, *Zc (field zone)*, *Pt (possession time)* and *Match Status*.

The model proposed for successful teams:

$$\begin{array}{l} \text{Pt}=\text{μ}+{\text{β}}_{1}\text{ Type of start}-up+{\text{β}}_{\text{2}}\text{ Intention}\\ +{\text{β}}_{3}Zc+{\text{β}}_{4}\text{ Match status}+{\text{β}}_{5}\text{ Type of start-up}:\\ \text{Intention: Zc:Match status} \end{array}$$

Results obtained from the variance analysis in successful teams are presented in Table 2.

**Table 2:** ANOVA results for successful teams.

	Df Sum	Sq Mean	Sq *F*	value	Pr(>*F*)
Type of start-up	1	1580	1580	6.638	0.01013^∗^
Intention	1	34303	34303	144.104	<2e-16^∗∗∗^
Zc	1	1872	1872	7.863	0.00515^∗∗^
Match Status	2	251	125	0.527	0.59046
Type of start-up: Intention	1	617	617	2.591	0.10783
Type of start-up: Zc	1	626	626	2.629	0.10528
Type of start-up: Match Status	2	534	267	1.121	0.32646
Intention: Zc	1	284	284	1.193	0.27503
Intention: Match Status	2	742	371	1.558	0.21104
Zc: Match Status	2	625	312	1.312	0.26977
Type of start-up: Intention: Zc	1	265	265	1.115	0.29131
Type of start-up: Intention: Match Status	2	698	349	1.466	0.23148
Type of start-up: Zc: Match Status	2	1645	823	3.456	0.03197 ^∗^
Intention: Zc: Match Status	2	496	248	1.043	0.35287
Type of start-up: Intention: Zc: Match status	2	894	447	1.879	0.15339
Residuals		938	223284	238	

*Significant codes: 0 “^∗∗∗^” 0.001 “^∗∗^” 0.01 “^∗^” 0.05 “.” 0.1 “ ”.*

Significant effects in this analysis were: the simple effects, *Type of start-up, Intention, Zc*. No significant second-order effects, and only a significant third-order effect *Type of star-up-Zc-Match Status.*

The transformation of the response variable made with the *Box–Cox* method, offers the best maximization of the *likelihood* profile, estimating the λ value that in this case is around 0.02020202.

This transformation allows to obtain a *Shapiro–Wilk* test of normality of W = 0.99669 with a *p-*value = 0.2821, verifying the normality test of the residuals in the model.

The ANOVA was again calculated with the transformation, and the following results were obtained (Table 3):

**Table 3:** ANOVA with transformation.

	Df Sum	Sq Mean	Sq F	Value	Pr(>*F*)
Type of start-up	1	10.6	10.56	19.654	1.04e-05^∗∗∗^
Intention	1	95.8	95.79	178.370	<2e-16^∗∗∗^
Zc	1	7.4	7.44	13.852	0.00021^∗∗∗^
Match Status	2	0.4	0.20	0.373	0.68898
Type of start-up: Intention	1	3.2	3.16	5.880	0.01550^∗^
Type of start-up: Zc	1	1.8	1.81	3.378	0.06638.
Type of start-up: Match Status	2	2.1	1.07	1.991	0.13717
Intention: Zc	1	0.4	0.37	0.690	0.40653
Intention: Match Status	2	0.5	0.25	0.465	0.62805
Zc: Match Status	2	0.4	0.21	0.396	0.67295
Type of start-up: Intention: Zc	1	0.1	0.13	0.245	0.60243
Type of start-up: Intention: Match Status	2	0.6	0.32	0.590	0.55468
Type of start-up: Zc: Match Status	2	3.9	1.97	3.659	0.02612^∗^
Intention: Zc: Match Status	2	0.2	0.12	0.225	0.79858
Type of start-up: Intention: Zc: Match status	2	0.8	0.39	0.730	0.48213
Residuals		938	503.8	0.54	

*Significant codes: 0 “^∗∗∗^” 0.001 “^∗∗^” 0.01 “^∗^” 0.05 “.” 0.1 “ ”.*

The significant effects in this analysis with the transformation applied were: simple effects, *Type of start-up, Intention, Zc*. Significant second-order effects, *Type of start-up-Intention; Type of start-up-Zc*. Finally, a third-order interaction *Type of start-up-Zc-Match Status* was significant.

### Main Effects in Successful Teams

**FIGURE 1:**
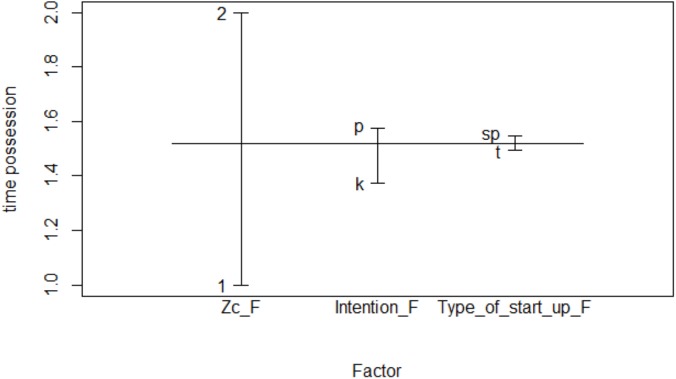
Main effects in successful teams.

The main effects of the three significant simple factors were represented, with their values related to possession time (Figure 1). In this way, possession time with respect to Type of start-up was obtained, and was slightly greater when the start is given in a set piece than in transition. With respect to the *Zc* a greater possession of the ball was observed in the offensive zone. Regarding the factor *Intention* of the observed team, when it recovers the ball, it was observed that the greatest possession time was given in an intention to progress with the ball *(p: progress*), and somewhat less when the intention was to preserve the ball (*k: keep*). The combination of the three factors with the maximum time of possession would be: in offensive zone, starting from a set pieces and with the intention of progressing the ball.

### Interactions in Successful Teams

**FIGURE 2:**
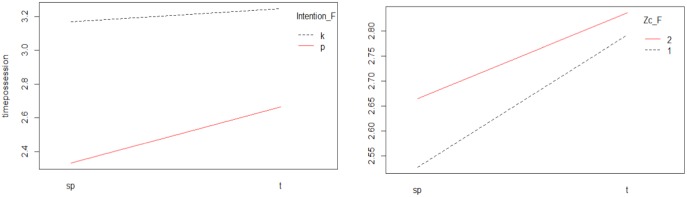
Interaction Type of start-up-Intention and Type of start-up-Zc.

The significant interactions of the model (*Type of start-up-Intention; Type of start-up-Zc)* were shown (Figure 2). In the case of the *Type of start-up-Intention* interaction, it could be seen how, both in the plays that started at set pieces and in transition, the greatest possession time occurred when the team intended to keep the ball.

In the *Type of start-up-Zc* interaction, a greater possession time was observed, both in the offensive zone, and the defensive zone, when the offensive phase was initiated by means of a transition.

### Successful Teams Predictions

Predictions of the significant interactions in the model were established, based on *possession time*, obtaining the prediction’s most adjusted values, accompanied by their confidence intervals (Table 3).

A first prediction was formed by the *Type of start-up-Intention* factors. The values of the categories of the beginning of the sequence were presented in relation to team intention. In Table 4 it can be observed, how a greater possession of the ball will be produced when the play begins by means of a transition.

**Table 4:** Predictions based on possession time.

Intention	Type of start-up	Fit	lwr	upr
Keep	Sp	3.168067	3.042380	3.293754
	T	3.248004	3.126791	3.369216
Progress	Sp	2.332448	2.245356	2.419540
	T	2.664055	2.592382	2.735727

**Zc**	**Type of start-up**	**Fit**	**lwr**	**upr**

Defensive zone	Sp	2.528168	2.412159	2.644176
	T	2.792668	2.696641	2.888695
Offensive zone	Sp	2.665467	2.560275	2.770659
	T	2.837030	2.743069	2.930992

A second prediction configured by the factors *Type of start-up-Zc*, indicates that there will always be greater possession of the ball, both in the defensive and offensive zones, if initiated by means of a transition.

For the prediction of three factors *Type of start-up-Zc-Match Status* the following situations were collected (Table 5):

**Table 5:** Three factor prediction.

Type of star-up	Set pieces		

**Zc**	**Match status**	**fit**	**lwr**	**Upr**
Defensive zone	Wn	2.331815	2.177045	2.486584
	Dr	2.712268	2.561506	2.863030
	Ls	2.520898	2.097975	2.943821
Offensive zone	Wn	2.571334	2.427036	2.715632
	Dr	2.746415	2.601379	2.891451
	Ls	2.730275	2.422766	3.037783

**Type of star-up**	**Dynamic transition**		

**Zc**	**Match status**	**fit**	**lwr**	**Upr**

Defensive zone	Wn	2.753987	2.620310	2.887663
	Dr	2.828626	2.698067	2.959185
	Ls	2.796901	2.470895	3.122908
Offensive zone	Wn	2.877259	2.758385	2.996132
	Dr	2.746526	2.594403	2.898650
	Ls	2.890032	2.631293	3.148770

If the play is started at set pieces actions, there will be a longer possession time with a tied score, both in the defensive and offensive zones.

In the case of a start with transition. There will be a longer possession time in the defensive zone with the score tied and in the offensive zone with the marker losing.

The model proposed for unsuccessful teams was the same as for successful ones:

$$\begin{array}{l} \text{Pt}=\text{μ}+{\text{β}}_{1}\text{ Type of start}-\text{up}\\ +{\text{β}}_{\text{2}}\text{ Intention}+{\text{β}}_{3}Zc+{\text{β}}_{4}\text{ Match Status}\\ +{\text{β}}_{5}\text{ Type of start-up}:\text{Intention: Zc: Match Status} \end{array}$$

Once the lack of normality adjustment to residuals was verified and the transformation of the explained variable was carried out applying the Box–Cox transformation, with a value λ = 0.1414141, which allows obtaining a Shapiro–Wilk coefficient. W = 0.9974, and a *p*-value = 0.1798, justifying the adjustment of the residuals of the model.

Table 6 shows the results obtained in the variance analysis, with the new adjustment.

**Table 6:** ANOVA results with transformed dependent variable, for non-successful teams.

	Df Sum	Sq Mean	Sq *F*	value	Pr(>*F*)
Type of start-up	1	0.7	0.66	0.644	0.422456
Intention	1	151.1	151.06	148.180	<2e-16^∗∗∗^
Zc	1	0.0	0.02	0.018	0.8903189
Match Status	2	17.7	8.84	8.671	0.000185^∗∗∗^
Type of start-up: Intention	1	5.7	5.72	5.613	0.018019^∗^
Type of start-up: Zc	1	10.3	10.34	10.140	0.001496^∗∗^
Type of start-up: Match Status	2	8.1	4.04	3.961	0.019340^∗^
Intention: Zc	1	1.9	1.85	1.819	0.177760
Intention: Match Status	2	33.0	16.52	16.200	1.19e-07^∗∗∗^
Zc: Match Status	2	0.9	0.44	0.436	0.646832
Type of start-up: Intention: Zc	1	1.0	0.99	0.975	0.323739
Type of start-up: Intention: Match Status	2	1.2	0.61	0.597	0.550505
Type of start-up: Zc: Match Status	2	10.1	5.06	4.966	0.007144^∗∗^
Intention: Zc: Match Status	2	0.6	0.30	0.298	0.742660
Type of start-up: Intention: Zc: Match status	2	2.5	1.25	1.222	0.294964
Residuals		993	1012.3	1.02	

*Significant codes: 0 “^∗∗∗^” 0.001 “^∗∗^” 0.01 “^∗^” 0.05 “.” 0.1 “ ”.*

The following significant values were observed: simple effects: *Intention (p < 0.001) y Match Status (p < 0.001)*. Second order effects: *Type of start-up- Intention (p < 0.05); Type of start-up-Zc (p < 0.01)*; *Type of start-up-Match Status (p < 0.05); Intention-Match Status (p < 0.001)*. Third order significant effect: *Type of start-up-Zc-Match Status (p < 0.01)*

### Main Effects for Unsuccessful Teams

**FIGURE 3:**
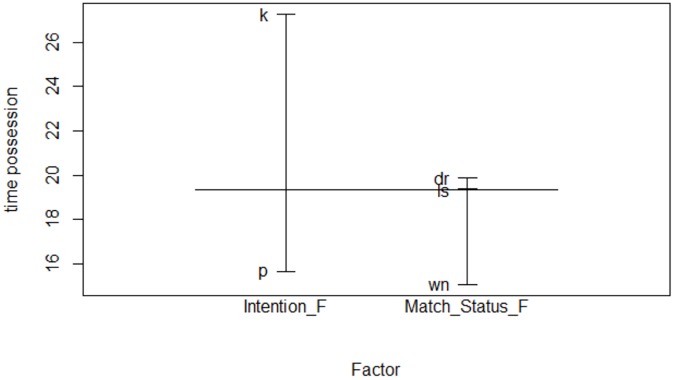
Main effects for unsuccessful teams.

The main effects were represented, of the two significant simple factors and their values related to the time of possession (Figure 3). In this way, it was observed that the greatest possession time with respect to the *Intention* factor occurs when there was an intention to keep the ball (*k: keep*), and much less when the intention was to progress (*p: progress*). In the factor score (*Match Status*), the greatest possession time was given when the team was losing, while the shortest possession time was given when the team was winning. The combination of the two factors indicate that the maximum possession time is achieved, based on an intention to keep the ball, when the team is losing.

### Unsuccessful Teams Interactions

**FIGURE 4:**
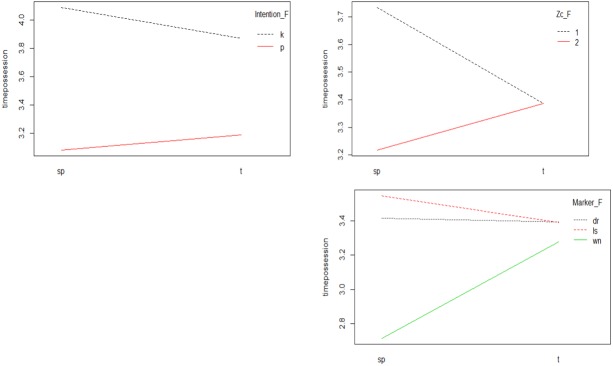
Interaction Type of start-up-Intention; Type of start-up-Zc and Interaction Type of star-up-Match Status.

Figure 4 shows the significant interactions of the model, according to the type of start-up. In the case of the *Type of start-up-Intention* interaction, it could be observed that, in both types of starts of the play, the longest possession time occurred when the intention was to keep the ball.

In the *Type of start-up-Zc* interaction, the longest possession time was given in the case of starting with a set piece in the defensive zone. In the case of start with transition, possession time was the same in both zones.

In the *Type of start-up-Match Status* interaction, it was observed how the greatest possession time occurred when the team started the play on set pieces and was losing. The shortest time of possession occurred, both in the start of set actions and transition, with the score in favor. With the score drawing there were hardly any differences in possession time regardless of the form of start of the play.

**FIGURE 5:**
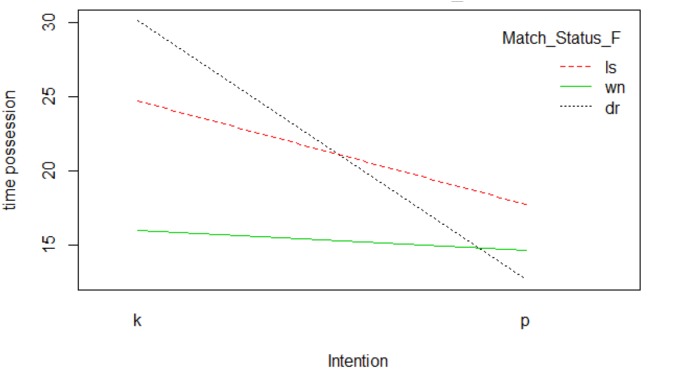
Interaction Intention-Match Status.

If we analyze the *Intention-Match Status* interaction, we can observe how two intersections occurred (Figure 5). The draw marker interacted with the winning and losing markers. This is because possession time with a draw score was much greater when the intention was to keep the ball, whereas when the intention was to progress it decreased considerably. When the score was favorable (winning) shorter possession times occurred, regardless of the team’s intention. With the score losing, the longest possession time occurred with the intention of keeping the ball with a descent when the intention was to progress.

### Unsuccessful Teams Prediction

Table 7 shows the results corresponding to the significant interactions with the Type of start-up. We can observe how a longer possession time will be produced when the play is initiated by means of a transition, whether it is intended to keep or progress.

**Table 7:** Predictions in function to Type of start-up.

Intention	Type of start-up	Fit	lwr	upr
Keep	Sp	3.080707	2.958752	3.202662
	T	3.187973	3.087256	3.288690
Progress	Sp	11.58845	9.752692	13.42420
	T	16.09780	14.587048	17.60855

**Zc**	**Type of start-up**	**fit**	**lwr**	**upr**

Defensive zone	Sp	3.735140	3.576295	3.893985
	T	3.386873	3.256824	3.516921
Offensive zone	Sp	3.218056	3.081412	3.354700
	T	3.386374	3.262865	3.509883

**Match status**	**Type of start-up**	**fit**	**lwr**	**upr**

Winning	Sp	2.714127	2.287977	3.140278
	t	3.279219	2.917061	3.641377
Drawing	Sp	3.416937	3.264049	3.569826
	T	3.395566	3.256741	3.534390
Lossing	Sp	3.549402	3.398372	3.700433
	T	3.392165	3.266919	3.517412

We can also observe how, in the defensive zone, greater possession will occur if it is started by means of a set pieces and, in the offensive zone, if it is initiated by means of a transition.

Finally, A greater possession time with the score winning will occur when the play is initiated by means of a transition. Both with the score losing and drawing, there will be a longer possession time when set pieces actions start.

In Table 8, we present the significant predictions based on Match status. If we analyze the predictions based on team intention and partial result we can see how, with the score drawing, there will be a longer possession time if you try to keep the ball and, with the result of losing, there will be a greater possession if you try to progress.

**Table 8:** Prediction according to tactical intention – marker.

Intention	Match status	fit	lwr	upr
Conservar	Wn	3.067949	2.607933	3.527966
	Dr	4.127844	3.980021	4.275666
	Ls	3.880861	3.698573	4.063149
Progresar	Wn	3.030887	2.725103	3.336672
	Dr	2.899664	2.776021	3.023308
	Ls	3.323472	3.221544	3.425401

For the prediction of three factors (*Type of start-up-ZC and Match Status*), the following situations may be possible (Table 9):

**Table 9:** Prediction field zone-type of start-up-marker.

Zc	Defensive zone		

**Type of start-up**	**Match status**	**fit**	**lwr**	**upr**
Sp	Wn	2.726720	2.146522	3.306918
	Dr	3.798074	3.594471	4.001677
	Ls	3.773970	3.561142	3.986798
T	Wn	3.147875	2.752974	3.542777
	Dr	3.503992	3.321563	3.686422
	Ls	3.325186	3.147818	3.502555

**Zc**	**Offensive zone**		

**Type of start-up**	**Match status**	**fit**	**lwr**	**upr**

Sp	Wn	2.707461	2.236707	3.178214
	Dr	3.091747	2.901043	3.282452
	Ls	3.399087	3.218663	3.579510
T	Wn	3.620713	3.056228	4.185197
	Dr	3.289762	3.109245	3.470280
	Ls	3.442400	3.286265	3.598534

In the defensive zone will be greater possession when the score is drawing, whether the play is initiated by means of a transition or set pieces.

There will be greater possession of the ball in offensive zone when the set piece starts with the score losing, and, if it starts with a transition, with the score winning.

## Discussion

Ball possession has been identified as a differentiating performance indicator between successful and unsuccessful teams (Grant et al., 1999a,b; Hook and Hughes, 2001; Jones et al., 2004; Bloomfield et al., 2005; Carling et al., 2005; Hughes and Franks, 2005; Casal et al., 2015, 2017). This work was proposed with the intention of discriminating possession time qualitative and quantitatively in successful and unsuccessful teams from a *mixed methods* perspective, to try to identify an effective ball possession model. The results have allowed us to identify significant differences between ball possession models of both groups of teams.

Specifically, based on the results of the simple effects, we have detected that in successful teams possession time is influenced by, the form of start of the offensive phase, the intention of the team once possession is recovered and possession zone. Successful teams are characterized by having longer possessions in the offensive zone when they start at set pieces actions and with the intention of progressing. On the other hand, in unsuccessful teams possession time of the offensive phase is influenced by team intention, once the possession of the ball has been recovered, and by the match status. In the latter case, our results corroborate those obtained in previous studies (Sasaki et al., 1999; James et al., 2004; Jones et al., 2004; Bloomfield et al., 2005; Lago-Peñas and Martín, 2007; Taylor et al., 2008; Lago-Peñas, 2009). These teams will have longer possessions when they are losing, results in line with some previous works (Sasaki et al., 1999; Jones et al., 2004; Bloomfield et al., 2005; Lago-Peñas and Martín, 2007; Lago-Peñas, 2009) and with the intention of keeping the ball. Of these results, perhaps the most significant is to indicate how the partial result, in successful teams, does not influence possession time. Indicating, in this case, that these teams do not vary their game model based on match status, while unsuccessful ones do, coinciding with the results of Bloomfield et al. (2005). These data differ from some of the previously mentioned works since, their results show the same pattern of ball possession, for both groups of teams, depending on the evolution of the match status. This circumstance may indicate an evolution of the game of successful teams toward more stable possession models, and less influenced by the evolution of the match status.

If we analyze the results obtained when studying the interaction of the different variables selected with possession time, we can observe how in the second order interactions there are also significant differences between both groups of teams. While successful teams are characterized by having longer possessions in the offensive zone, when they start possession through a transition, unsuccessful teams have longer possessions in the defensive zone, initiating the attack through set pieces ball actions and, above all, when they are losing. We cannot compare these results with previous works, since we have not found any study that performs a multivariate analysis with the indicators selected here. Some previous works (Casal et al., 2017) have also found that successful teams are characterized by possessions of longer duration in the offensive zone and unsuccessful ones, on the contrary, in the defensive zone. This can be explained because successful teams are supposed to have a higher technical-tactical level, and are able to overcome the greater defensive pressure and accumulation of players near the opposing goal and, on the contrary, unsuccessful teams will only be able of maintaining possession in those areas of lower defensive pressure that, in general, are close to your goal.

Finally, observing the data obtained in third-order interactions, which will allow us to make predictions about how possession time of the team will be, according to the relationships between the selected variables. In this case, we can check how the main differences between both teams occur in the following cases:

Successful teams will always have greater possession time, both in defensive and offensive zone in the event that the play is initiated by means of a transition. These data are in line with what was previously stated when finding that successful teams have longer possession times than unsuccessful ones, indicating that higher-level teams try to control the game, and take the initiative, through ball possession, helped by the high individual performances of their players. In the case of unsuccessful teams, if the offensive phase starts on set pieces actions, the greatest possession will occur in the defensive zone and, if the play is initiated by means of a transition, in the offensive zone. This can be explained because, in a set pieces action, the opposing team has enough time to organize defensively and, therefore, lower level teams will have greater difficulties to advance toward areas closer to the opponent’s goal. On the contrary, if they start the offensive phase after a recovery of the ball, it may be easier for them to progress to more advanced zones, due to the defensive disorganization of the opposing team, since this is in an open disposition, with greater inter and intra-line space.

If we take into account the type of start-up and the match status, successful teams will produce their greatest possession with a score draw and starting the play by means of a set pieces ball action. This data shows, once again, the control of the game performed by higher level teams, maintaining possession of the ball. In the case of unsuccessful teams, if they are winning, they will have greater possession initiating the play through a transition. As we discussed earlier, in this circumstance of the game, the opposing team will perform a defensive pressure, because of their need to score as much, and the lower level of unsuccessful teams will not allow them to maintain possession for a long time unless they initiate the attack through a dynamic transition, without leaving time for the rival team to organize defensively. If they find themselves losing or drawing, the possession will be longer if the play starts at set pieces actions. In this case, the rival team does not have the need to press defensively, which will facilitate the team possession.

If we consider the type of start-up, the match status and field zone. In this case, we see how successful teams will always have longer possessions initiating the offensive plays by means of a transition and this possession will be more extensive in the defensive zone if they are drawing and in the offensive zone if they are winning. In the first case, it can be considered a normal behavior, since not having the need to score so much, can give up on counterattacks and its main objective can be focused on keeping the ball, as a defensive method. The second behavior is somewhat contradictory, since, if they are winning, it is normal for the opposing team to perform defensive pressure and this pressure will be greater near their goal, so maintaining possession in this zone will be more difficult that to do it near the own goal, where the pressure of the adversary team is smaller. This behavior could be explained by the need to keep the ball as far as possible from the own goal, to avoid a possible chance of an opponent’s goal, in the case of losing the ball to the opponent. On the other hand, unsuccessful teams, in the case of being drawing, will always have more extensive possessions in the defensive zone, regardless of the type of start of the play. In addition, in spite of being able to have the will to progress toward the rival goal, it lacks the technical-tactical mechanisms necessary for it, hence that it passes great moments of the game in the initial gestation phase of the offensive game. Adversary teams, in this situation, do not have the need to quickly recover the possession of the ball, and may allow it to be in the power of the opposing team, but away from their own goal. For these teams possessions will be of greater duration in the offensive zone, losing, and initiating the play by means of a set pieces action and winning, by means of a transition. In the first case, the need to score as much, will provoke a more advanced defensive pressure and possession of the ball closer to the area of the opposing team’s goal. The second situation has already been explained previously, in this case the defensive pressure of the rival team will only allow to have the ball a minimum of time in control, until they are able to make a counterattack.

Based on the results obtained, we can prove how our hypothesis regarding possession analysis from the mixed methods perspective was confirmed, which would allow us to differentiate the possessions of successful and unsuccessful teams and describe a more effective possession style. The application of the results of this study in the field of intervention will affect the tactical-strategic aspects of the team’s game. This knowledge will allow the elaboration of intervention strategies to optimize team possession. However, the results of this work cannot be generalized to all matches and competitions, because only national teams have been analyzed and in a specific competition. As some previous works indicate (James et al., 2004; Bloomfield et al., 2005; Tucker et al., 2005; Lago-Peñas and Martín, 2007; Lago-Peñas, 2009; Collet, 2013), the type of competitions influences team possession, therefore, it will be necessary to continue investigating with different samples that cover different competitions to try to generalize the results and try to identify which are the key elements that differentiate or characterize the offensive possessions of successful teams, with the objective of trying to identify a more effective possession model.

## Conclusion

This work has been carried out with the intention of identifying which are the performance indicators that influence possession time in elite soccer teams, check if these indicators differ between successful and unsuccessful teams and finally, identify a more effective possession model.

It has been possible to verify the existence of differences between the performance indicators that influence possession time between successful and unsuccessful teams. Specifically, in successful teams possession time is influenced by: *Type of start-up, intention and field zone.* On the other hand, possession time of unsuccessful teams is determined fundamentally by *intention and match status.* We have also noted how the phase of the tournament in which the match is played, the match period, the interaction context and the number of passes do not influence team possession time. Finally, the models to execute the offensive phase that guarantee a greater possession of the ball have also been different for both groups of teams.

## Data Availability

The datasets generated for this study are available on request to the corresponding author.

## Author Contributions

CC developed the project, review the literature, and wrote the manuscript. JL was responsible for performed analysis and the method section. MA wrote part of the manuscript and revised the content critically and RM revised the content and supervised the drafting of the manuscript.

## Conflict of Interest Statement

The authors declare that the research was conducted in the absence of any commercial or financial relationships that could be construed as a potential conflict of interest.
